# Intrahepatic infiltration of activated CD8^+^ T cells and mononuclear phagocyte is associated with idiosyncratic drug-induced liver injury

**DOI:** 10.3389/fimmu.2023.1138112

**Published:** 2023-03-01

**Authors:** Hyun Yang, Ji Won Han, Jae Jun Lee, Ahlim Lee, Sung Woo Cho, Pu Reun Rho, Min-Woo Kang, Jeong Won Jang, Eun Sun Jung, Jong Young Choi, Pil Soo Sung, Si Hyun Bae

**Affiliations:** ^1^ The Catholic University Liver Research Center, College of Medicine, The Catholic University of Korea, Seoul, Republic of Korea; ^2^ Division of Hepatology, Department of Internal medicine, College of Medicine, Eunpyeong St. Mary’s Hospital, The Catholic University of Korea, Seoul, Republic of Korea; ^3^ Division of Hepatology, Department of Internal medicine, College of Medicine, Seoul St. Mary’s Hospital, The Catholic University of Korea, Seoul, Republic of Korea; ^4^ Department of Hospital Pathology, College of Medicine, Eunpyeong St. Mary’s Hospital, The Catholic University of Korea, Seoul, Republic of Korea

**Keywords:** drug-induced liver injury, T cell, mononuclear phagocyte, flow cytometry, steroid

## Abstract

**Background:**

Idiosyncratic drug-induced liver injury (DILI) is caused by the interplay among drugs, their metabolites, and the host immune response. The characterization of infiltrated immune cells in the liver may improve the understanding of the pathogenesis of idiosyncratic DILI. This study investigated the phenotypes and clinical implications of liver-infiltrating immune cells in idiosyncratic DILI.

**Methods:**

From January 2017 to June 2021, 53 patients with idiosyncratic DILI who underwent liver biopsy were prospectively enrolled in this study. Immunohistochemical staining and flow cytometry analyses were performed on the biopsy specimens. Serum levels of CXC chemokine ligand 10 (CXCL10) and soluble CD163 were measured. A multivariate cox proportional hazards model was used to evaluate predictors of DILI resolution within 30 days.

**Results:**

The numbers of intrahepatic T cells and mononuclear phagocytes were positively correlated with serum levels of total bilirubin, alanine aminotransferase (ALT), and the model of end-stage liver disease score. The frequency of activated CD8+ T cells among liver-infiltrating CD8+ T cells in DILI livers was higher than that in healthy livers. Notably, the percentages of activated intrahepatic CD8+ T cells and mononuclear phagocytes in DILI livers showed a positive correlation with ALT. Additionally, serum CXCL10 level was positively correlated with intrahepatic T cell infiltration and ALT, and soluble CD163 level was positively correlated with intrahepatic mononuclear phagocyte infiltration and ALT. Thirty-six patients (70.6%) were treated with steroids. In multivariate analysis, total bilirubin and steroid use independently influenced DILI resolution within 30 days.

**Conclusions:**

Activated CD8+ T cells and mononuclear phagocyte are associated with liver injury caused by drugs. Therefore, we suggest that steroids are a potential treatment option for idiosyncratic DILI.

## 1 Introduction

Drug-induced liver injury (DILI) is defined as liver damage caused by prescribed or over-the-counter drugs, herbs, health food, dietary supplements, folk remedies, or other causes resulting in abnormal liver tests or liver dysfunction with the reasonable exclusion of other etiologies (1–4). DILI is a major cause of acute liver failure in the US (5). Although determining the actual incidence of DILI is difficult, the annual DILI incidence has been reported to be 2.7 cases per 100,000 persons in the US (6) and 12 hospitalized cases per 100,000 persons in the Republic of Korea (4).

DILI is traditionally classified as an intrinsic or idiosyncratic type (2, 3). Idiosyncratic DILI occurs only in susceptible individuals and exhibits variable onset latency (2, 3). The pathogenesis of idiosyncratic DILI is not well understood; however, innate and adaptive immune responses may play a critical role in liver damage during DILI (2, 7, 8). Interactions between drugs or metabolites and human leukocyte antigen (HLA) are hypothesized to elicit an adaptive immune response (7, 8). Drug-induced sterile inflammation and drug-modified self-antigen-associated immune cell activation and recruitment have been proposed as possible immune-mediated mechanisms of idiosyncratic DILI (8). The histopathologic findings of idiosyncratic DILI show the infiltration of various immune cells (9, 10). Therefore, theoretically, corticosteroids may be effective for treating idiosyncratic DILI by reducing immune-mediated liver injury (11).

In clinical practice, liver biopsy in patients with DILI is useful for differential diagnosis, when features of autoimmune hepatitis are present, and for assessing the severity of DILI (2, 12). Furthermore, the characterization of infiltrated immune cells in the liver may improve the understanding of idiosyncratic DILI pathogenesis. In an immunohistochemical study, the predominant infiltration of leukocytes by cytotoxic T cells has been shown in idiosyncratic DILI (10). However, infiltrated immune cells have not been characterized by functional phenotyping combined with immunohistochemical staining in idiosyncratic DILI.

This study investigated the phenotypes of infiltrating immune cells in idiosyncratic DILI and evaluated the correlations between liver-infiltrating immune cells and clinical parameters. We also evaluated predictors of DILI resolution.

## 2 Materials and methods

### 2.1 Patients

This prospective study was conducted in Seoul St. Mary’s Hospital and Eunpyeng St. Mary’s Hospital of the Catholic University of Korea from January 2017 to June 2021. Idiosyncratic DILI diagnosis was based on the medication history of patients after the exclusion of other etiologies including viral hepatitis, alcoholic liver disease, autoimmune hepatitis, ischemic hepatitis, or extrahepatic obstruction of the bile duct. Patients with idiosyncratic DILI that underwent liver biopsy were enrolled in this study. Inclusion criteria based on a previous prospective DILI study were as follows (13): 1) serum aspartate aminotransferase (AST) or alanine aminotransferase (ALT) > five times the upper limit of normal (ULN) (or pretreatment baseline if baseline level was elevated) on two separate occasions; 2) serum alkaline phosphatase (ALP) > two times the ULN (or pretreatment baseline if baseline level was elevated) on two separate occasions; 3) serum total bilirubin > 2.5 mg/dL; or 4) international normalized ratio (INR) > 1.5. Exclusion criteria were as follows: 1) suspected acetaminophen overdose, 2) preexisting viral hepatitis, 3) preexisting immune-mediated liver disease, and 4) preexisting alcoholic liver disease.

This study was approved by the Institutional Review Board (IRB) of the Catholic University of Korea (PC19OESI0012 and KC20OESI0102). All enrolled subjects provided written informed consent. Liver tissue and peripheral blood from healthy subjects were collected in accordance with the IRB approval.

### 2.2 Clinical information

We assessed the baseline characteristics, including laboratory tests at the time of liver biopsy. Serum biochemical parameters included total bilirubin, AST, ALT, ALP, gamma-glutamyl transpeptidase, albumin, creatinine, and prothrombin time. We also performed tests for anti-nuclear, anti-mitochondrial, anti-smooth muscle, and anti-liver-kidney microsomal type 1 antibodies. The model of end-stage liver disease (MELD) was calculated according to the published formula (14).

The types, causality, classification of the causative agent, and severity of DILI were assessed in previous studies. The types of liver injuries were classified based on the R value, which is the ratio of ALT/ULN to ALP/ULN. Hepatocellular type was defined as R ≥ 5, cholestatic type as R ≤ 2, and mixed type as 2 < R < 5 (3). Causality was assessed using the Roussel Uclaf Causality Assessment Method (RUCAM) scale. According to this scale, patients were grouped into likelihood levels of ‘excluded’ (score ≤ 0), ‘unlikely (1, 2)’, ‘possible’ (3–5), ‘probable’ (6–8), and ‘highly probable’ (> 8) (15). The causative agents of DILI were classified into the following categories as previously described: ‘medication’, ‘herb’ ‘health food or dietary supplement’, ‘folk remedy’, ‘combined’, and ‘others’ (4). The severity of DILI was assessed based on the severity criteria proposed by the International DILI Working Group in 2011 (16).

Hepatic histopathological features were recorded for all liver biopsies. Fibrosis stages and necro-inflammatory grades were scored as previously described (17, 18).

### 2.3 Resolution of DILI and patient follow-up

Resolution of DILI was defined as the return of patient serum AST, ALT, ALP, and total bilirubin levels to normal. Follow-up duration was calculated from the date of DILI diagnosis to the date of resolution or the last follow-up visit. Patients with abnormal serum ALT, AST, ALP, or total bilirubin were censored at the date of their last recorded follow-up.

### 2.4 Immunohistochemistry

Cross-sections (5 µM) of paraffin-embedded blocks were moved to salinized glass slides. The sections were deparaffinized using xylene and rehydrated using a series of graded alcohols. Antigen retrieval was performed using a microwave vacuum histoprocessor (RHS-1; Milestone, Bergamo, Italy) by heating the samples in 0.01 M citrate buffer (pH 6.0) for 20 min to a final temperature of 121 °C. The sections were incubated for 10 min with hydrogen peroxide (3%) in methanol to prevent endogenous peroxidase activity. The slides were incubated with anti-CD3 (Abcam, Cambridge, UK) and anti-CD68 (clone: KP1; Dako, Carpinteria, CA, USA) antibodies. After washing, an EnVision+ system HRP-labelled polymer (Dako) was used at 24°C for 5 min. The slides were treated with 3,3’-diaminobenzidine for 5 min and then counterstained with hematoxylin.

**Figure 1:**
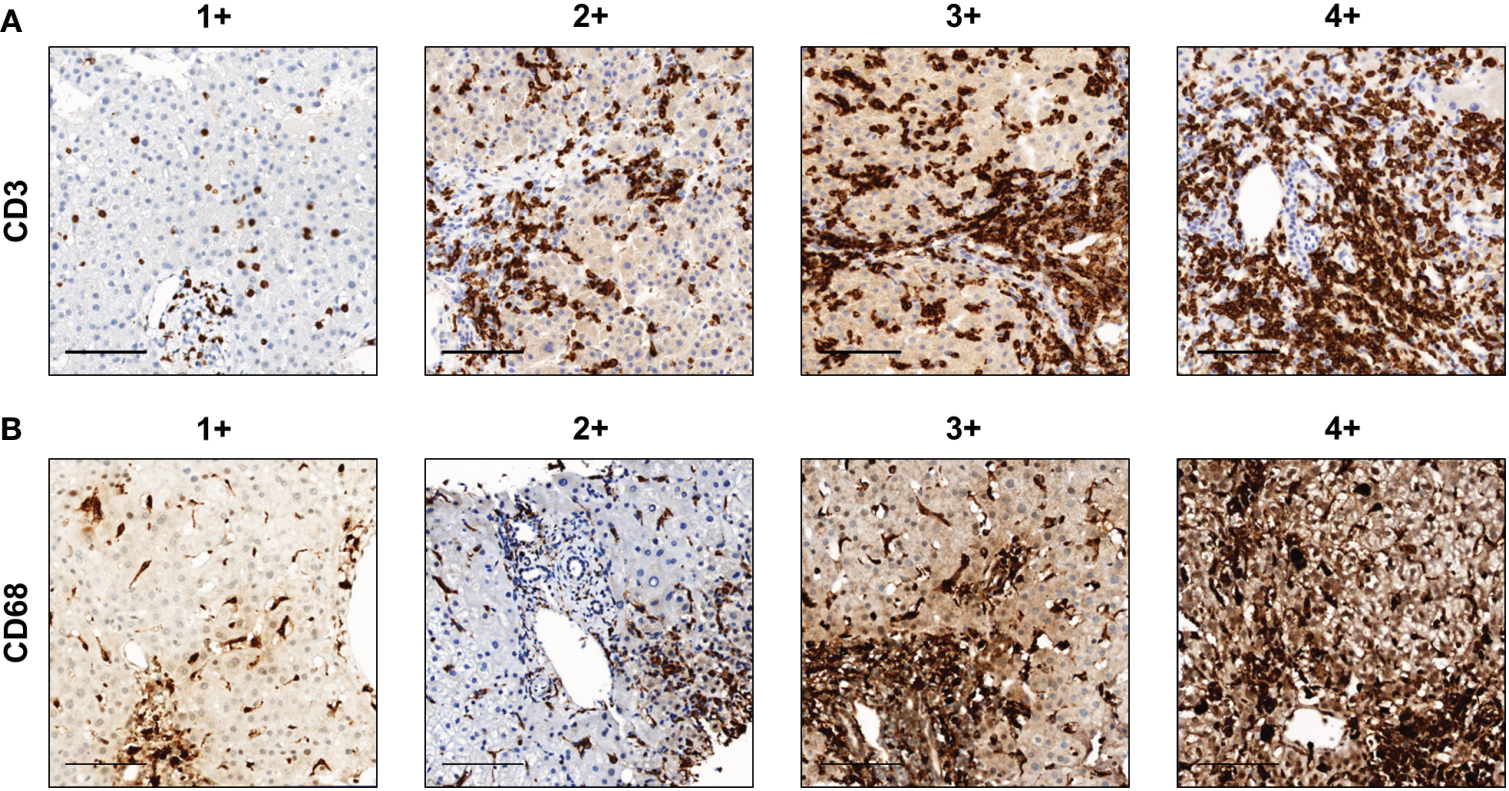
Representative images of the amount of CD3+ and CD68+ cell infiltration. **(A)** CD3 and **(B)** CD68. Scale bar, 100 μm. The degree of immune cell infiltration was analyzed in more than five randomly selected high-power (×200) microscopic fields. The amounts of CD3+ and CD68+ cell infiltration were classified into the following five groups: 0, 1+, 2+, 3+, and 4+ when the percentages of stained cells were 0, >0–10, >10–30, >30–50, and >50%, respectively.

The degree of immune cell infiltration was analyzed in more than five randomly selected high-power (×200) microscopic fields. The amount of CD3- and CD68-positive cell infiltration was classified into the following five groups: 0, 1+, 2+, 3+, and 4+ when the percentages of stained cells were 0, >0–10, >10–30, >30–50, and >50%, respectively (**Figure 1**). The scoring was confirmed by an experienced pathologist (ESJ).

### 2.5 Flow cytometry

Subjects included in this study were undergoing percutaneous liver biopsy for diagnostic purposes. A cell strainer (FALCON, 352350) was used to mash the biopsy samples. After that, the samples were digested by collagenase (0.05%)/Hyaluronidase (1000 U/mL) and DNase (5 U/mL) to obtain a cell suspension. Subsequently, the supernatant was removed, and the pellet was treated with an RBC lysis buffer as previously described (19).

Multicolor flow cytometry was performed using the following commercially available antibodies: phycoerythrin (PE)-conjugated anti-human CD38 and CD80, PerCP-Cy5.5-conjugated anti-human CD16, PE-cyanine 7 (Cy7)-conjugated anti-human NKG2D (BioLegend, San Diego, CA, USA), allophycocyanin (APC)-Cy7-conjugated anti-human HLA-DR, APC-conjugated anti-human CD69, V450-conjugated anti-human CD8, V500-conjugated anti-human CD4, and PerCP-Cy5.5-conjugated anti-human CD3 (BD Biosciences, San Jose, CA, USA). LIVE-DEAD Fixable Violet Dead Cell Stain Kit (Thermo Fisher Scientific, L34955) was used to discriminate dead cells from live cells. Data were recorded using an LSR Fortessa, Canto II instrument (BD Biosciences) and analyzed using the FlowJo software (TreeStar, Ashland, OR, USA). Intracellular cytokine staining was performed using a previously described protocol (20).

### 2.6 Enzyme-linked immunosorbent assay

ELISAs for Human CXC Chemokine Ligand 10 (CXCL10) and soluble CD163 were performed using commercially available kits according to the manufacturer’s instructions (R&D Systems Ltd, Minneapolis, MN, USA).

### 2.7 Statistical analyses

Continuous data are expressed as mean ± standard deviation or median (range) and categorical data are expressed as number or percentage. Categorical variables between groups were compared using the Chi-square or Fisher’s exact tests and continuous variables were compared using independent *t* or Mann–Whitney test. Correlations between variables were analyzed using Spearman or Pearson coefficients. A logistic regression model was used to identify an independent predictor for DILI resolution within 30 days. For univariate analysis, *p-*value < 0.2 was considered statistically significant to be included in the multivariate analysis. For multivariate analysis, *p-*value < 0.05 was considered statistically significant. Statistical analyses were conducted using GraphPad Prism (v.7.0, San Diego, CA, USA) and SPSS (v.20.0, Chicago, IL, USA).

## 3 Results

### 3.1 Patient characteristics and histopathological findings

**Table 1** shows the baseline characteristics of 53 patients enrolled in this study. The cohort was predominantly female (71.7%), with a mean age of 53.3 ± 15.6 years. Fourteen patients (26.4%) were related to ‘medication’ and another 14 (26.4%) were related to ‘herb’. The RUCAM scores ranged between 5–14 points with an average of 7.8. Forty-two patients (79.2%) had a highly probable causal relationship between the suspected agent and liver injury. Forty-seven patients (88.7%) were classified under hepatocellular-type injury. Severity grades 2, 3, and 4 were present in 10 (19.6%), 35 (68.6%), and six (11.8%) patients, respectively. Detailed information about the cases and causative drugs is described in **Supplementary Table 1**.

**Table 1:** Baseline characteristics.

	DILI (n=53)
Mean Age (years)	53.3 ± 15.6
Female (%)	38 (71.7)
Causality (%)	
- Medications	14 (26.4)
- Herb	14 (26.4)
- Health foods or dietary supplement	10 (18.9)
- Folk remedies	8 (15.1)
- Mixed	7 (13.2)
RUCAM score	7.8 ± 2.2
Type of liver injury (%)	
- Hepatocellular	47 (88.7)
- Mixed	5 (9.4)
- Cholestatic	1 (1.9)
White blood cell count (10^3^/µL)	5.5 ± 2.0
Hemoglobin (g/dL)	12.7 ± 1.6
Platelet count (10^3^/µL)	221.5 ± 80.4
Total bilirubin (mg/dL)	6.5 ± 5.7
Direct bilirubin (mg/dL)	4.7 ± 4.2
Alkaline phosphatase (IU/L)	156.8 ± 106.9
Gamma glutamyl transpeptidase (IU/L)	283.3 ± 242.2
Aspartate aminotransferase (IU/L)	693.8 ± 492.9
Alanine aminotransferase (IU/L)	1023.7 ± 721.1
Total protein (mg/dL)	6.4 ± 0.7
Albumin (mg/dL)	3.7 ± 0.5
INR	1.1 ± 0.2
Creatinine (mg/dL)	0.7 ± 0.2
ANA positive (%)	35 (66.0)
Anti-smooth muscle Ab positive (%)	3 (5.8)
Anti-liver-kidney-microsomal Ab positive (%)	0 (0.0)
Immunoglobulin G (mg/dL)	1337.7 ± 315.3
Child-Pugh Class (%)	
- A	21 (39.6)
- B	31 (58.5)
- C	1 (1.9)
MELD score	13.2 ± 5.1
Severity* (%)	
- Grade 2	10 (19.6)
- Grade 3	35 (68.6)
- Grade 4	6 (11.8)

Data are n (%) or mean ± SD. Categorical variables between groups were compared using the Chi-square or Fisher’s exact tests and continuous variables were compared using an independent t or Mann-Whitney U test.

INR, international normalized ratio; ANA, anti-nuclear antibody; Ab, antibody; MELD, the model of end-stage liver disease.

*The severity of DILI was assessed in accordance with the severity criteria for DILI proposed by the international DILI working group in 2011.

The subjects showed various levels of Knodell’s histological activity index (mean value 7.1 ± 3.4, range 1–15) and low grades of fibrosis (F0 and F1) (**Table 2**).

**Table 2:** Histopathological findings.

	DILI (n=53)
Knodell’s fibrosis grade (%) 0/1	42 (79.2)/11 (20.8)
Knodell’s histological activity index	7.1 ± 3.4
CD3 (%) 1+/2+/3+/4+	9 (17.0)/26 (49.1)/13 (24.5)/5 (9.4)
CD68 (%) 1+/2+/3+/4+	15 (28.3)/13 (24.5)/16 (30.2)/9 (17.0)

Data are n (%) or mean ± SD.

### 3.2 Outcome of DILI

Fifty patients (94.3%) were followed up until DILI resolution. No cases of death or liver transplantation were observed during follow-up. Among the 50 patients, the median duration until DILI resolution was 42.0 (11−287) days. Fourteen patients (28.0%) experienced DILI resolution within 30 days.

Thirty-seven patients (69.8%) were treated with steroids. The median daily dose of corticosteroid was 13.3 mg of prednisolone or equivalent. The median duration of corticosteroid treatment was 30 (7–107) days. No patients experienced relapse after the cessation of steroid therapy. In multivariate analysis, steroid use [odds ratio (OR) = 7.736, 95% confidence interval (CI), 1.223–48.936; *p* = 0.030] was an independent favorable factor for DILI resolution within 30 days and higher total bilirubin level (OR = 0.848, 95% CI, 0.733–0.975; *p* = 0.021) was an independent unfavorable factor for DILI resolution within 30 days (**Table 3**).

**Table 3:** Factors that influence the resolution of DILI within 30 days according to univariate and multivariate analyses.

	Univariate analysis		Multivariate analysis		
	OR	*p*	OR	95% CI	*p*
Age	0.966	0.115	0.961	0.917-1.007	0.092
Male	0.620	0.521			
Hepatocellular-type injury*	0.750	0.757			
Total bilirubin (mg/dL)	0.883	0.070	0.848	0.736-0.977	0.023
AST (IU/L)	0.999	0.224			
ALT (IU/L)	1.000	0.382			
ALP (IU/L)	1.002	0.516			
CD3 positive cell infiltration >50%	1.833	0.533			
CD68 positive cell infiltration >50%	1.364	0.695			
ANA positive	1.413	0.614			
Use of steroid	3.000	0.192	6.426	1.011-40.839	0.049

A logistic regression model was used to identify an independent predictor for DILI resolution within 30 days.

DILI, drug-induced liver injury; AST, aspartate aminotransferase; ALT, alanine aminotransferase; ALP, alkaline phosphatase; ANA, anti-nuclear antibody; OR, odds ratio; CI, confidence interval.

* Hepatocellular-type injury was defined as R value ≥ 5.

### 3.3 Infiltration of CD3^+^ T cells and CD68^+^ mononuclear phagocytes in the DILI livers

**Figure 2:**
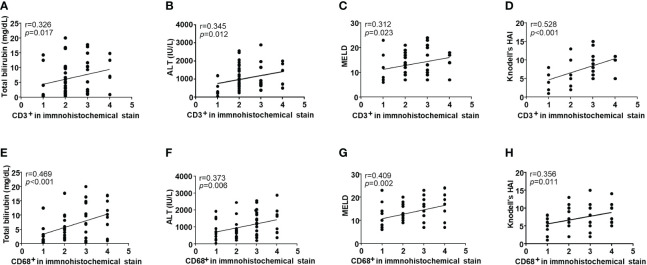
Correlation with clinical parameters and the infiltration of intrahepatic immune cells in drug-induced liver injury (DILI) patients. Intrahepatic CD3^+^ cell infiltration showed a significant positive correlation with **(A)** total bilirubin, **(B)** ALT, **(C)** MELD, and **(D)** Knodell’s histological activity index (r = 0.326, 0.345, 0.312, and 0.528, respectively, *p* < 0.05). Intrahepatic CD68^+^ cell infiltration showed a significant positive correlation with **(E)** total bilirubin, **(F)** ALT, **(G)** MELD, and **(H)** Knodell’s histological activity index (r = 0.469, 0.373, 0.409, and 0.356, respectively, *p* < 0.05). Correlations between variables were analyzed using Spearman or Pearson coefficients. ALT, alanine aminotransferase; MELD, the model of end-stage liver disease; HAI, histological activity index.

To analyze T cell and mononuclear phagocytes infiltration in DILI livers, we performed immunohistochemistry of liver tissues from 53 patients with DILI. **Figure 1** shows representative images of the amount of (A) CD3+ and (B) CD68+ cell infiltration in DILI livers. T cell (CD3^+^) and mononuclear phagocyte (CD68^+^) infiltration was observed in all subjects (**Table 2**). CD3^+^ cell infiltration showed a significant positive correlation with total bilirubin, ALT, MELD, and Knodell’s histological activity index (r = 0.326, 0.345, 0.312, and 0.528, respectively, *p* < 0.05) (**Figures 2A–D**). CD68^+^ cell infiltration showed a significant positive correlation with total bilirubin, ALT, MELD, and Knodell’s histological activity index (r = 0.469, 0.373, 0.409, and 0.356, respectively, *p* < 0.05) (**Figures 2E–H**). There were no significant differences in CD3^+^ and CD68^+^ cell infiltration according to ANA positivity (**Supplementary Table 2**).

### 3.4 Phenotypes of infiltrative immune cells in the DILI livers

To analyze the phenotypes of infiltrative immune cells in the DILI liver, we performed flow cytometry using liver tissue from 24 patients with DILI (**Supplementary Figure 1**). Only patients who agreed to FACS analysis of liver tissue were analyzed consecutively. The baseline characteristics of patients who underwent FACS analysis are shown in **Supplementary Table 3**.

**Figure 3:**
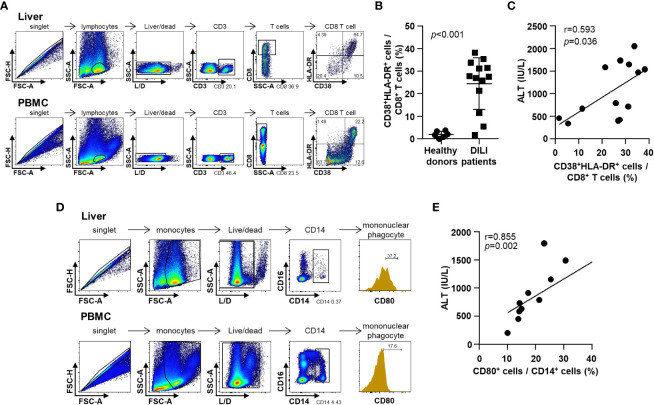
Phenotypes of infiltrative immune cells in the DILI livers. **(A)** Representative flow cytometry result of intrahepatic T cell activation in a patient with DILI. **(B)** Frequency of activated (CD38^+^HLA-DR^+^) CD8^+^ T cells in patients with DILI was significantly higher than that in healthy donors (*p* < 0.001). **(C)** Percentage of activated (CD38^+^HLA-DR^+^) CD8^+^ T cells in DILI livers was positively correlated with serum ALT (r = 0.593, *p* = 0.036). **(D)** Representative flow cytometry result of intrahepatic mononuclear phagocyte activation in a patient with DILI. **(E)** Percentage of activated (CD80^+^) CD14^+^ mononuclear phagocytes in the DILI livers was positively correlated with serum ALT (r = 0.855, *p* = 0.002). Correlations between variables were analyzed using Spearman or Pearson coefficients. PBMC, peripheral blood mononuclear cell; ALT, alanine aminotransferase.

Representative dotplots and gating strategies for liver samples and corresponding peripheral blood samples are presented as **Figures 3A, D**. To be consistent with all the gatings, at times, we used peripheral blood samples to differentiate positivity and negativity of each intensity value of fluorescence.

First, we examined the activation status of liver-infiltrating CD8^+^ T cells in patients with DILI (**Figures 3A–C**). The frequency of activated (CD38^+^HLA-DR^+^) CD8^+^ T cells in patients with DILI was significantly increased higher than that in healthy donors (**Figure 3B**). The percentage of activated (CD38^+^HLA-DR^+^) CD8^+^ T cells in the DILI livers was positively correlated with serum ALT (r = 0.593, *p* = 0.036) (**Figure 3C**).

Second, we examined the activation status of liver-infiltrating mononuclear phagocytes in patients with DILI (**Figure 3D**). The percentage of activated (CD80^+^) CD14^+^ mononuclear phagocytes in the DILI liver was positively correlated with serum ALT (r = 0.855, *p* = 0.002) (**Figure 3E**).

### 3.5 Correlations with clinical parameters and immune cell-related cytokines

**Figure 4:**
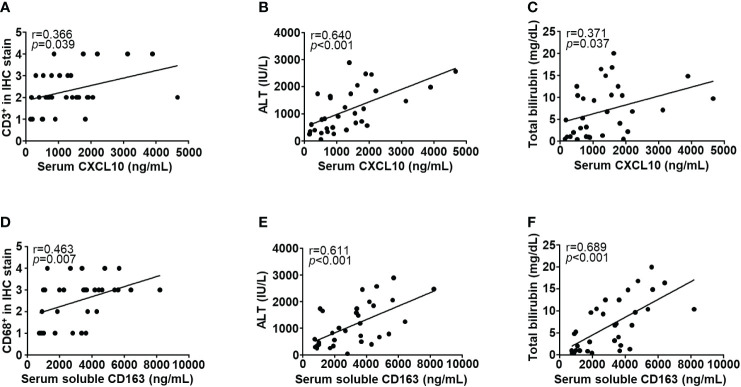
Correlations with clinical parameters and immune cell-related cytokines. Serum CXCL10 level was positively correlated with **(A)** intrahepatic CD3^+^ cell infiltration, **(B)** ALT, and **(C)** total bilirubin (r = 0.366, 0.640, and 0.371, respectively, *p* < 0.05). Soluble CD163 level in serum was positively correlated with **(D)** intrahepatic CD68^+^ cell infiltration, **(E)** ALT, and **(F)** total bilirubin (r = 0.463, 0.611 and 0.689, respectively, *p* < 0.05). Correlations between variables were analyzed using Spearman or Pearson coefficients. IHC, immunohistochemistry; ALT, alanine aminotransferase; CXCL10, CXC chemokine ligand 10.

To investigate the cytokine response of immune cells, we performed ELISA for serum CXCL10 and soluble CD163 from 32 patients with DILI (**Supplementary Figure 1**). Serum CXCL10 level was positively correlated with CD3^+^ cell infiltration, ALT and total bilirubin (r = 0.366, 0.640 and 0.371, respectively, *p* < 0.05) (**Figures 4A–C**). Soluble CD163 level in serum was positively correlated with CD68^+^ cell infiltration, ALT and total bilirubin (r = 0.463, 0.611 and 0.689, respectively, *p* < 0.05) (**Figures 4D–F**).

## 4 Discussion

In this study, we characterized liver-infiltrating immune cells and investigated the correlation between immunological and clinical parameters in patients with idiosyncratic DILI. In multivariate analysis, total bilirubin level was identified as a significant risk factor for DILI, and steroid use was a significant favorable factor for DILI resolution within 30 days. The degrees of T cell and mononuclear phagocyte infiltration showed a positive correlation with the deterioration of liver function in patients with idiosyncratic DILI. Additionally, the activation status of liver-infiltrating T cells and mononuclear phagocytes showed a positive correlation with ALT levels, and serum cytokines associated with these immune cells showed a positive correlation with total bilirubin and ALT levels.

Innate and adaptive immunity can play important roles in the immune-mediated mechanism of idiosyncratic DILI. Our study showed that abundant T cells and mononuclear phagocytes infiltrated DILI livers, showing the important role of these immune cells in idiosyncratic DILI. The amount of immune cell infiltration was positively correlated with the deterioration of liver function. The degrees of T cell and macrophage infiltrates are considerably high in patients with altered hepatic function (10, 21). Furthermore, we observed that activated T cells, activated mononuclear phagocytes, and related cytokines were correlated with the degree of liver damage.

Although the exact role of T cells in idiosyncratic DILI is not well-known, drugs, drug-induced neoantigens, or drug metabolites may trigger T cell activation directly or indirectly (7). Interferon-independent robust CXCL10 production (22) and T cell receptor-independent activation of CD8^+^ T cell (23) have been suggested as mechanisms for innate-like cytotoxicity in patients with hepatitis A. In addition, innate-like bystander-activated CD38^+^HLA-DR^+^CD8^+^ T cells are known to play a pathogenic role in patients with chronic hepatitis C (24). An increased proportion of activated helper and cytotoxic T cells has been observed in the peripheral blood of acute idiosyncratic patients with DILI (21). Therefore, both innate and adaptive immune responses involving T cells may play an important role in idiosyncratic DILI.

The role of mononuclear phagocytes in idiosyncratic DILI is also not clear; however, it is assumed to be related to the secretion of migration-inhibitory factors of macrophages (25) or the lipopolysaccharide protein pathway (26). Similar to our findings, the amount of CD68^+^ cell infiltration in DILI liver and plasma soluble CD163 level have been found to be correlated with the severity of liver injury, and the lipopolysaccharide protein-related pathway has been suggested as a cause (26).

Given the importance of the immune-mediated mechanism of idiosyncratic DILI, immunosuppression with corticosteroids could theoretically be effective in treating idiosyncratic DILI (11). The use of corticosteroids in patients with DILI has significantly reduced recovery time (27–29). Considering clinical practice guidelines of the American Association for the Study of Liver Disease and the European Association for the Study of Liver Disease for DILI, steroids can be considered as a treatment option for DILI or autoimmune hepatitis-like DILI caused by immune checkpoint inhibitors (1, 2). One reason for the good response rate to corticosteroids in this study may be that 66.0% of patients had anti-nuclear antibody positivity, which can be interpreted as an autoimmune feature.

The key strengths of this study include the demonstration of a significant positive correlation between liver injury and the amount of immune cell infiltration using immunohistochemistry and the proportion of activated infiltrating immune cells using flow cytometry in inflamed DILI livers. To the best of our knowledge, it is one of the largest studies using liver tissues from patients with idiosyncratic DILI. Furthermore, these results are consistent with respect to the correlation between liver injury and serum cytokine levels associated with immune cells. These findings will be helpful to understand underlying immune mechanisms in idiosyncratic DILI.

This study also has several limitations. First, as this is not a randomized controlled trial, presenting a clear rationale for steroid treatment in DILI is challenging. However, a randomized controlled trial for steroid treatment in DILI has not been performed because designing it is difficult, considering the various causes and clinical courses of DILI. Nevertheless, well-designed randomized controlled trials for specific immune cell-related idiosyncratic DILI are needed. Second, the number of patients included in this study is insufficient to generalize the results. Therefore, the results require further confirmation through an independent cohort. Third, the dosage and duration of steroids varied as they were determined by individual clinicians. Fourth, depending on the degree of consent of each patient, the patients who underwent FACS analysis and those who underwent ELISA did not overlap. Using inconsistent samples may introduce selection bias. Fifth, the infiltration of T cells and macrophages is not specific to DILI (10). Further studies comparing immune cell infiltration in DILI with that in other etiologies are needed. Finally, we could not elucidate the exact immunologic mechanism of idiosyncratic DILI. However, our group recently developed a DILI mouse model using thioacetamide and observed the intrahepatic infiltration of activated mononuclear phagocytes in this model (30). Further *in vivo* and *ex vivo* studies using this model will help understand the detailed immunologic mechanism of idiosyncratic DILI.

In conclusion, our results indicate that hepatic T cell and mononuclear phagocyte infiltration along with proportions of their activated phenotype and related cytokines correlated with the degree of liver injury in patients with idiosyncratic DILI. Therefore, we suggest that T cells and mononuclear phagocytes play critical roles in idiosyncratic DILI. Furthermore, our findings suggest that steroids may be a potential treatment option for idiosyncratic DILI.

## Data availability statement

The raw data supporting the conclusions of this article will be made available by the authors, without undue reservation.

## Ethics statement

The studies involving human participants were reviewed and approved by the Institutional Review Board (IRB) of the Catholic University of Korea. The patients/participants provided their written informed consent to participate in this study. Written informed consent was obtained from the individual(s) for the publication of any potentially identifiable images or data included in this article.

## Author contributions

HY (conceptualization, methodology, formal analysis, investigation, resources, data curation, writing – original draft, writing – review & editing, visualization, project administration, funding acquisition), JH (resources, writing – review & editing, funding acquisition), JJ (resources), AL (resources), SWC (investigation), PR (investigation), M-WK (investigation and writing – review), JJ (resources), ESJ (conceptualization, resources, data curation), JC (resources), PS (conceptualization, methodology, formal analysis, investigation, resources, data curation, writing – original draft, writing – review & editing, visualization, supervision, project administration, funding acquisition), SB (conceptualization, methodology, resources, data curation, writing – review & editing, supervision, project administration, funding acquisition). All authors contributed to the article and approved the submitted version.
