# Corrigendum: Intrahepatic infiltration of activated CD8^+^ T cells and mononuclear phagocyte is associated with idiosyncratic drug-induced liver injury

**DOI:** 10.3389/fimmu.2023.1201876

**Published:** 2023-04-25

**Authors:** Hyun Yang, Ji Won Han, Jae Jun Lee, Ahlim Lee, Sung Woo Cho, Pu Reun Rho, Min-Woo Kang, Jeong Won Jang, Eun Sun Jung, Jong Young Choi, Pil Soo Sung, Si Hyun Bae

**Affiliations:** ^1^ The Catholic University Liver Research Center, College of Medicine, The Catholic University of Korea, Seoul, Republic of Korea; ^2^ Division of Hepatology, Department of Internal Medicine, College of Medicine, Eunpyeong St. Mary’s Hospital, The Catholic University of Korea, Seoul, Republic of Korea; ^3^ Division of Hepatology, Department of Internal Medicine, College of Medicine, Seoul St. Mary’s Hospital, The Catholic University of Korea, Seoul, Republic of Korea; ^4^ Department of Hospital Pathology, College of Medicine, Eunpyeong St. Mary’s Hospital, The Catholic University of Korea, Seoul, Republic of Korea

**Keywords:** drug-induced liver injury, T cell, mononuclear phagocyte, flow cytometry, steroid

In the published article, there was an error in **Figure 3A** as published. The X-axis and Y-axis labels of the sixth panel from the top and sixth panel from the bottom in **Figure 3A** were incorrectly displayed as “FSC-H” and “SSC-A”, respectively.

The correct labels for these panels should be “HLA-DR” and “CD38”, respectively. The corrected **Figure 3A** and its caption appear below.

**Figure 3:**
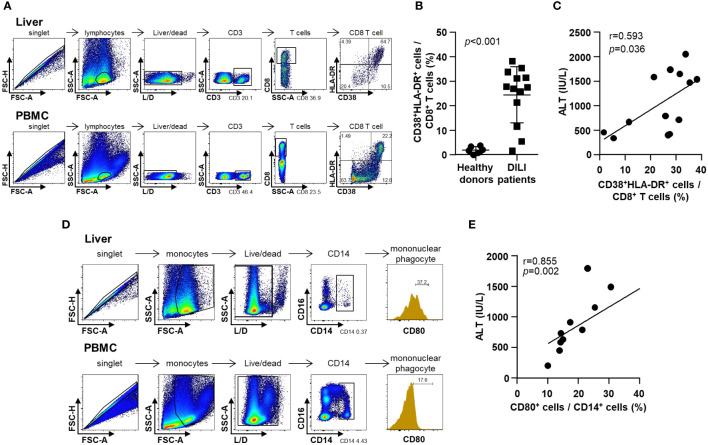
Phenotypes of infiltrative immune cells in the DILI livers. **(A)** Representative flow cytometry result of intrahepatic T cell activation in a patient with DILI. **(B)** Frequency of activated (CD38^+^HLA-DR^+^) CD8^+^ T cells in patients with DILI was significantly higher than that in healthy donors (p < 0.001). **(C)** Percentage of activated (CD38^+^HLA-DR^+^) CD8^+^ T cells in DILI livers was positively correlated with serum ALT (r = 0.593, *p* = 0.036). **(D)** Representative flow cytometry result of intrahepatic mononuclear phagocyte activation in a patient with DILI. **(E)** Percentage of activated (CD80^+^) CD14^+^ mononuclear phagocytes in the DILI livers was positively correlated with serum ALT (r = 0.855, *p* = 0.002). Correlations between variables were analyzed using Spearman or Pearson coefficients. PBMC, peripheral blood mononuclear cell; ALT, alanine aminotransferase.

The authors apologize for this error and state that this does not change the scientific conclusions of the article in any way. The original article has been updated.

