# Supplementary material for: Intrahepatic infiltration of activated CD8+ T cells and mononuclear phagocyte is associated with idiosyncratic drug-induced liver injury
Source: Front Immunol. 2023 Mar 1;14:1138112. doi: 10.3389/fimmu.2023.1138112 (PMC10014460; doi:10.3389/fimmu.2023.1138112)
Supplement: Supplementary file 1 [file DataSheet_1.docx]

***Supplementary Materials***

Intrahepatic Infiltration of Activated CD8^+^ T Cells and Mononuclear Phagocyte is Associated with Idiosyncratic Drug-induced Liver Injury

Hyun Yang^1,2^, Ji Won Han^1,3^, Jae Jun Lee^1^, Ahlim Lee^1,2^, Sungwoo Cho^1^, Pu Reun Rho^1^, Min-Woo Kang^1^, Jeong Won Jang^1,3^, Eun Sun Jung^4^, Jong Young Choi^1,3^, Pil Soo Sung^1,3*^, Si Hyun Bae^1,2*^

*** Correspondence:** Pil Soo Sung: pssung@catholic.ac.kr, Si Hyun Bae: baesh@catholic.ac.kr

^1^The Catholic University Liver Research Center, College of Medicine, The Catholic University of Korea, Seoul, 06591, Republic of Korea

^2^Division of Hepatology, Department of Internal medicine, College of Medicine, Eunpyeong St. Mary’s Hospital, The Catholic University of Korea, Seoul, 03312, Republic of Korea

^3^Division of Hepatology, Department of Internal medicine, College of Medicine, Seoul St. Mary’s Hospital, The Catholic University of Korea, Seoul, 06591, Republic of Korea

^4^Department of Hospital Pathology, College of Medicine, Eunpyeong St. Mary’s Hospital, The Catholic University of Korea, Seoul, 03312, Republic of Korea

**A Table of Contents**

**Supplementary Figures**

Supplementary Figure 1. The patient flowchart.

**Supplementary Tables**

Supplementary Table 1. Causative drugs and characteristics of DILI.

Supplementary Table 2. Comparison of immune cell infiltration according to ANA positivity.

Supplementary Table 3. Baseline characteristics of the patients who underwent FACS analysis or not.


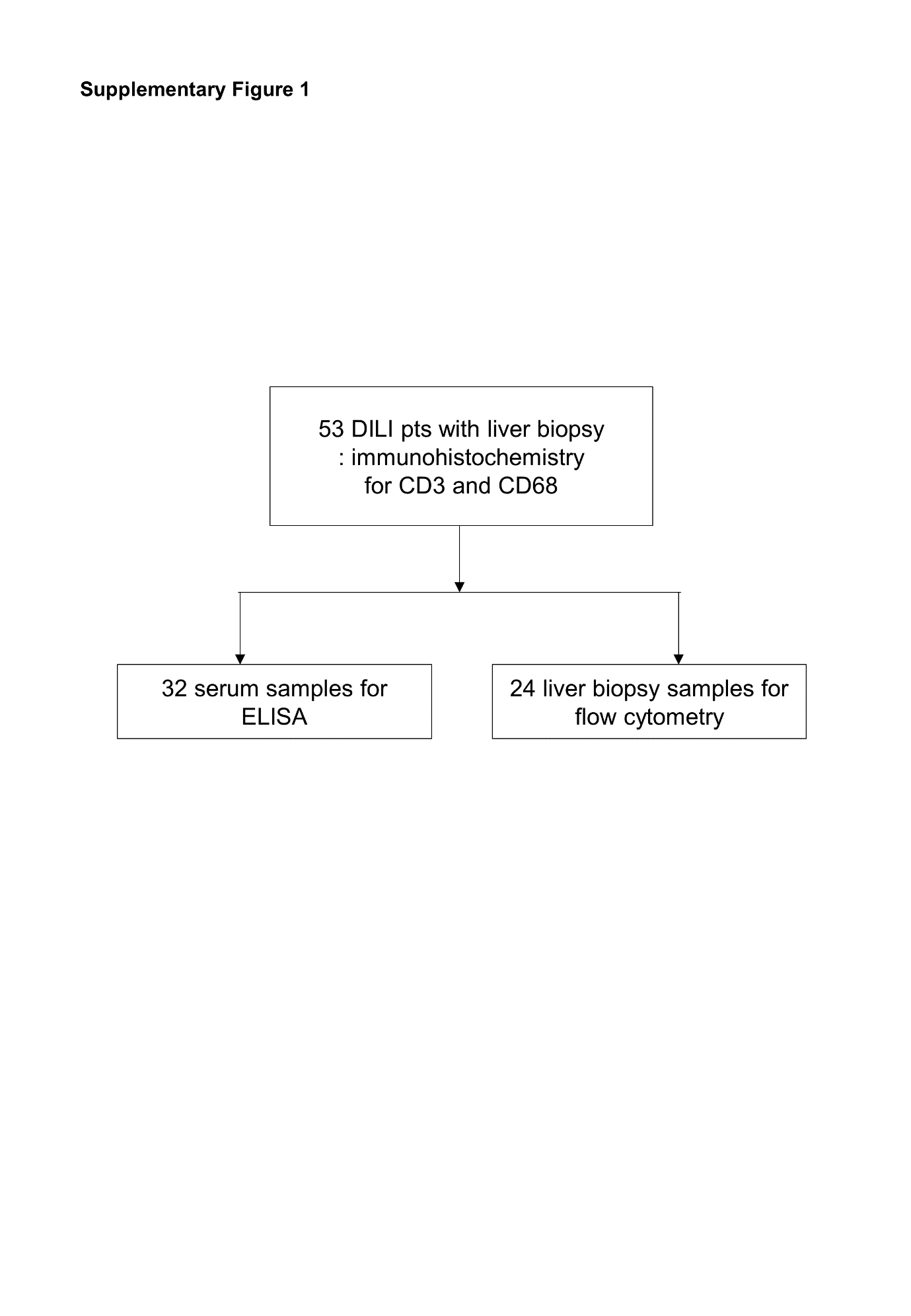
**Supplementary Figure 1.** The patient flowchart. DILI, drug-induced liver injury.

**Supplementary Table 1.** Causative drugs and characteristics of DILI.

| Patient Number | Causative drugs | Dose (mg/day) | Time to onset of DILI (days) | Maximum ALT (IU/L) | Description of the histology |
| --- | --- | --- | --- | --- | --- |
| 1 | *Helianthus tuberosus* | N/A | 30 | 2284 | Acute hepatitis |
| 2 | Isoniazid | 300 | 150 | 1200 | Acute hepatitis |
| 3 | Berries of *Panax ginseng* | N/A | 60 | 605 | Chronic hepatitis |
| 4 | Gefitinib | 250 | 90 | 2295 | Acute hepatitis |
| 5 | Herbal medications* | N/A | 150 | 939 | Chronic hepatitis |
| 6 | *Fallopia multiflora* extract | N/A | 30 | 1497 | Acute hepatitis |
| 7 | Jujube and burdock root-containing herbal decoction and ibuprofen | 400** | 20 | 1240 | Acute hepatitis |
| 8 | Black ginseng | N/A | 30 | 341 | Acute hepatitis |
| 9 | Herbal medications | N/A | 30 | 1887 | Acute hepatitis |
| 10 | *Ganoderma lucidum* | N/A | 30 | 1424 | Acute hepatitis |
| 11 | Zaltoprofen | 400 | 180 | 120 | Chronic cholestatic hepatitis |
| 12 | *Opuntia ficus-indica* extract | N/A | 180 | 336 | Chronic hepatitis |
| 13 | Red ginseng | N/A | 3 | 3322 | Acute hepatitis with eosinophilic infiltration |
| 14 | *Leonurus japonicas* | N/A | 3 | 2186 | Acute hepatitis |
| 15 | *Brassica oleracea var. capitate* and *Perilla frutescens* juice | N/A | 360 | 977 | Chronic hepatitis |
| 16 | *Pueraria montana var. lobate* | N/A | 30 | 2637 | Chronic hepatitis |
| 17 | *Brassica oleracea var. capitate* extract | N/A | 30 | 3144 | Acute hepatitis |
| 18 | Quetiapine | 50 | 90 | 520 | Chronic cholestatic hepatitis |
| 19 | Berries of *Euterpe Oleracea* and *Oscillatoria spirulina* extract | N/A | 90 | 446 | Acute hepatitis |
| 20 | Herbal medications | N/A | 60 | 2785 | Acute cholestatic hepatitis |
| 21 | Herbal medications, *Helianthus tuberosus* and *Momordica charantia* | N/A | 20 | 1485 | Chronic hepatitis |
| 22 | *Morinda citrifolia* juice | N/A | 30 | 1178 | Chronic cholestatic hepatitis |
| 23 | *Eleutherococcus sessiliflorus* juice | N/A | 90 | 1342 | Acute hepatitis |
| 24 | Gongjin-dan (Herbal medications) | N/A | 60-70 | 244 | Chronic hepatitis |
| 25 | Phenytoin | 200 | 7 | 557 | Acute hepatitis |
| 26 | Herbal medications | N/A | 5 | 1014 | Acute hepatitis |
| 27 | Ibuprofen | 1200 | 3 | 3778 | Acute hepatitis |
| 28 | Methylphenidate | 10 | 30 | 1621 | Acute hepatitis |
| 29 | Herbal medications | N/A | 30 | 3322 | Acute hepatitis |
| 30 | *Ganoderma lucidum* | N/A | 7 | 966 | Acute hepatitis |
| 31 | Cefaclor | 750 | 8 | 596 | Acute hepatitis |
| 32 | Herbal medications | N/A | 30 | 2199 | Acute hepatitis |
| 33 | Cefaclor | 750 | 7 | 496 | Chronic hepatitis |
| 34 | Nilotinib | 300-400 | 120-150 | 484 | Acute hepatitis |
| 35 | Herbal medications | N/A | 90 | 648 | Acute hepatitis |
| 36 | Herbal medications | N/A | 60 | 2345 | Acute hepatitis with eosinophilic infiltration |
| 37 | Cefaclor | 750 | 10 | 570 | Acute cholestatic hepatitis |
| 38 | Doxycycline | 200 | 10 | 299 | Acute hepatitis |
| 39 | Herbal medications and albendazole | 400*** | 20 | 1734 | Acute hepatitis with eosinophilic infiltration |
| 40 | Red ginseng | N/A | 10 | 1843 | Acute hepatitis |
| 41 | Herbal medications | N/A | 14 | 949 | Acute hepatitis |
| 42 | Albendazole | 400 | 7 | 823 | Acute hepatitis |
| 43 | Red ginseng | N/A | 180 | 1025 | Chronic hepatitis |
| 44 | Herbal medications and red ginseng | N/A | 30 | 2671 | Acute hepatitis |
| 45 | Herbal medication and *Pueraria montana var. lobata* | N/A | 30 | 1491 | Acute hepatitis |
| 46 | Herbal medications | N/A | 20 | 1401 | Acute hepatitis |
| 47 | Antler | N/A | 15 | 734 | Acute hepatitis with eosinophilic infiltration |
| 48 | Herbal medications | N/A | 45 | 4068 | Acute hepatitis |
| 49 | Herbal medications | N/A | 20 | 1535 | Acute hepatitis |
| 50 | Herbal medications | N/A | 20 | 1239 | Acute hepatitis |
| 51 | Herbal medications and antler | N/A | 20 | 943 | Acute hepatitis |
| 52 | Herbal medications and *Pueraria montana var. lobate* | N/A | 7 | 1795 | Acute hepatitis with eosinophilic infiltration |
| 53 | Herbal medications and *Oscillatoria spirulina* | N/A | 60 | 205 | Acute cholestatic hepatitis |

Abbreviations: DILI, drug-induced liver injury; ALT, alanine aminotransferase; N/A, not applicable

*Herbal medications: medications prescribed and compounded by a doctor of oriental medicine

** dose of ibuprofen

*** dose of albendazole

**Supplementary Table 2. Comparison of immune cell infiltration according to ANA positivity.**

|  | **ANA negative** | **ANA positive** | ***p*** |
| --- | --- | --- | --- |
|  | **(N=18)** | **(N=35)** |  |
| CD3 positive cells infiltration >50% | 3 (16.7%) | 2 (5.7%) | 0.426 |
| CD68 positive cells infiltration >50% | 1 (5.6%) | 8 (22.9%) | 0.229 |

Data are n (%). The categorical variables between groups were compared using the Chi-square or Fisher’s exact tests. Abbreviations: ANA, anti-nuclear antibody.

**Supplementary Table 3. Baseline characteristics of the patients who underwent FACS analysis or not**

|  | **FACS group** | **Non-FACS group** | ***p*** |
| --- | --- | --- | --- |
|  | **(N=24)** | **(N=29)** |  |
| Mean Age (years) | 52.0 ± 14.7 | 54.4 ± 16.4 | 0.573 |
| Female (%) | 18 (75.0) | 20 (69.0) | 0.858 |
| Causality (%) |  | | 0.167 |
| - Medications | 7 (29.2) | 7 (24.1) |  |
| - Herb | 9 (37.5) | 5 (17.2) |  |
| - Health foods or dietary supplement | 2 ( 8.3) | 8 (27.6) |  |
| - Folk remedies | 2 ( 8.3) | 6 (20.7) |  |
| - Mixed | 4 (16.7) | 3 (10.3) |  |
| RUCAM score | 8.7 ± 2.1 | 8.6 ± 1.4 | 0.882 |
| Type of liver injury (%) |  | | 0.280 |
| - Hepatocellular | 22 (91.7) | 25 (86.2) |  |
| - Mixed | 1 ( 4.2) | 4 (13.8) |  |
| - Cholestatic | 1 ( 4.2) | 0 ( 0.0) |  |
| White blood cell count (10^3^/µL) | 5.3 ± 1.8 | 5.7 ± 2.1 | 0.419 |
| Haemoglobin (g/dL) | 12.8 ± 1.2 | 12.6 ± 1.8 | 0.666 |
| Platelet count (10^3^/µL) | 209.6 ± 73.4 | 231.4 ± 85.8 | 0.331 |
| Total bilirubin (mg/dL) | 6.6 ± 5.8 | 6.4 ± 5.7 | 0.896 |
| Direct bilirubin (mg/dL) | 4.7 ± 4.1 | 4.7 ± 4.4 | 0.940 |
| Alkaline phosphatase (IU/L) | 195.8 ± 122.4 | 124.5 ± 80.7 | 0.019 |
| Gamma glutamyl transpeptidase (IU/L) | 324.4 ± 295.1 | 249.2 ± 186.5 | 0.286 |
| Aspartate aminotransferase (IU/L) | 709.3 ± 512.6 | 680.9 ± 484.7 | 0.837 |
| Alanine aminotransferase (IU/L) | 993.0 ± 558.5 | 1049.0 ± 841.5 | 0.773 |
| Total protein (mg/dL) | 6.4 ± 0.7 | 6.3 ± 0.6 | 0.609 |
| Albumin (mg/dL) | 3.8 ± 0.5 | 3.6 ± 0.5 | 0.136 |
| INR | 1.1 ± 0.2 | 1.1 ± 0.2 | 0.661 |
| Creatinine (mg/dL) | 0.7 ± 0.2 | 0.7 ± 0.3 | 0.459 |
| ANA positive (%) | 14 (58.3) | 21 (72.4) | 0.432 |
| Anti-smooth muscle Ab positive (%) | 1 ( 4.3) | 2 ( 6.9) | 1.000 |
| Anti-liver-kidney-microsomal Ab positive (%) | 0 (0.0) | 0 (0.0) |  |
| Immunoglobulin G (mg/dL) | 1292.4 ± 275.1 | 1373.6 ± 344.4 | 0.362 |
| Child-Pugh Class (%) |  | | 0.413 |
| - A | 8 (33.3) | 13 (44.8) |  |
| - B | 16 (66.7) | 15 (51.7) |  |
| - C | 0 ( 0.0) | 1 ( 3.4) |  |
| MELD score | 13.2 ± 4.7 | 13.2 ± 5.6 | 0.986 |
| Severity* (%) |  | | 0.409 |
| - Grade 2 | 1 ( 4.2%) | 0 ( 0.0%) |  |
| - Grade 3 | 20 (83.3%) | 27 (93.1%) |  |
| - Grade 4 | 3 (12.5%) | 2 ( 6.9%) |  |

Data are n (%) or mean ± SD. The categorical variables between groups were compared using the Chi-square or Fisher’s exact tests, and the continuous variables were compared using an independent t or Mann-Whitney U tests.

Abbreviations: INR, international normalised ratio; ANA, anti-nuclear antibody; Ab, antibody; MELD, the model of end-stage liver disease.

*The severity of DILI was assessed in accordance with the severity criteria for DILI proposed by the international DILI working group in 2011.
